# Steroid Pulse Therapy as a Treatment for Patients With COVID-19 Pneumonia at an Intensive Care Unit: A Single-Center Retrospective Observational Study

**DOI:** 10.7759/cureus.36386

**Published:** 2023-03-20

**Authors:** Hiromu Okano, Ryota Sakurai, Tsutomu Yamazaki

**Affiliations:** 1 Emergency and Critical Care Medicine, National Hospital Organization Yokohama Medical Center, Yokohama, JPN; 2 Department of Epidemiology and Social Medicine, Graduate School of Public Health, International University of Health and Welfare, Tokyo, JPN

**Keywords:** pneumoniae, steroid pulse therapy, methylprednisolone, intensive care unit, covid-19

## Abstract

Background: Evidence supporting the use of steroid pulse therapy in severely ill patients with coronavirus disease 2019 (COVID-19) pneumonia is lacking. Few studies have evaluated the efficacy of high-dose (1000 mg/day) methylprednisolone (mPSL), which is commonly used in Japan.

Aim: This study aimed to compare the clinical outcomes with and without steroid pulse therapy (mPSL 1000 or 500 mg/day for three days) in patients with COVID-19 pneumonia, admitted to an intensive care unit (ICU).

Methods: Study design was retrospective observational study. The inclusion criterion was severe to critically ill adult patients with COVID-19 pneumonia requiring ICU admission. The exclusion criteria were as follows: patients (1) with a “Do not attempt to resuscitate” order; (2) with a “Do not intubate” order; or (3) admitted to the ICU owing to other infectious diseases were excluded. Treatment strategy was as follows: Patients were divided into two groups: steroid pulse therapy (Group P) and steroids without pulse therapy (Group NP). Group P received mPSL 1000 or 500 mg/day on ICU days 1-3, and Group NP received dexamethasone 6.6 mg or mPSL 1 or 2 mg/kg/day. The primary outcome was 28-day mortality.

Results: We enrolled 82 patients. Out of 70 who met the inclusion criteria, 48 and 22 were included in Groups P and NP, respectively. No difference in 28-day survival was observed between the Groups P and NP (log-rank P=0.11). After adjusting for covariates (age, sex, interleukin-6 level, and acute physiology and chronic health evaluation II score on ICU admission) using a multivariate Cox proportional hazard model, treatment with steroid pulse therapy significantly improved 28-day mortality (hazard ratio, 0.14; 95% confidence interval, 0.02-0.86; P=0.03).

Conclusion: Steroid pulse therapy may improve the 28-day mortality in patients with COVID-19 pneumonia in the ICU.

## Introduction

Guidelines for the treatment of coronavirus disease 2019 (COVID-19) pneumonia have been significantly revised over the past few years [1]. For example, remdesivir is recommended for hospitalized patients with severe COVID-19 pneumonia, who are not on mechanical ventilation because some data suggest that it may reduce the time to recovery and the risk of mechanical ventilation [1,2]. The current Japanese guidelines also recommend corticosteroids for patients with COVID-19 pneumonia who require oxygen [3]. However, the optimal dose and duration of corticosteroid administration remain unclear. Three randomized controlled trials (RCTs) reported improved outcomes following steroid pulse therapy for COVID-19 pneumonia [4-6]. In contrast, other RCTs reported no efficacy of steroid pulse therapy [7,8].

The Japanese COVID-19 guidelines [3] do not provide recommendations for steroid pulse therapy. This recommendation was developed based on the findings of two low-quality RCTs [4,8], one of which [4] evaluated a corticosteroid dose different from the dose commonly used in Japan. However, the efficacy of steroid pulse therapy has not yet been fully investigated. In a previous study, in Japan, Okano et al. [9] reported the usefulness of steroid pulse therapy only in patients who were intubated.

Respiratory failure requiring intensive care unit (ICU) admission includes not only patients requiring intubation but also patients receiving noninvasive respiratory support, such as nasal high-flow oxygen and patients receiving high-concentration oxygen. Therefore, this study aimed to compare the clinical outcomes of patients with COVID-19 pneumonia admitted to an ICU between those treated with steroid pulse therapy and those treated without pulse therapy (non-pulse therapy).

## Materials and methods

Study design and setting

This is a single-center, retrospective, observational study. This study was approved by the Ethics Committees of the National Hospital Organization Yokohama Medical Center (No. 2021-14), where this study was conducted, and International University School of Health and Welfare Graduate School of Public Health (No. 21-Ig-216).

Patients

All adult patients with COVID-19 admitted to the ICU at the National Hospital Organization Yokohama Medical Center between April 1, 2020, and September 15, 2021, were enrolled.

Inclusion criteria

The inclusion criteria were as follows: (1) age ≥18 years with a (2) microbiologically confirmed COVID-19 diagnosis and respiratory failure (ratio of arterial oxygen partial pressure to fractional inspired oxygen (PaO2/FiO2) < 300 or oxygen saturation < 90%) on admission to the ICU. We included patients with "Severe" and "Critical" illness according to the WHO severity classification [1].

Diagnosis of COVID-19

Detection of severe acute respiratory syndrome coronavirus-2 (SARS-CoV-2) in nasopharyngeal or oropharyngeal swabs using polymerase chain reaction testing (bioMerieux Japan Ltd., Tokyo, Japan) was considered as microbiologically confirmed COVID-19.

Diagnosis of COVID-19 pneumonia

COVID-19 pneumonia was defined as a confirmed COVID-19 infection with pneumonia on computed tomography (CT).

Exclusion criteria

Individuals were excluded if they were patients: (1) with a “Do not attempt to resuscitate” order, (2) with a “Do not intubate” order, or (3) admitted to the ICU owing to other infectious diseases.

Treatment strategy

After assessment by several intensivists, patients with type H or L CT images (based on the classification by Gattinoni et al. [10] at presentation) were assigned to Groups P and NP, respectively. Based on a previous research [11], after admission to the ICU, patients in Group P received steroids according to the following protocol: 1000 or 500 mg/day methylprednisolone (mPSL) on ICU days 1-3. Thereafter, the dose was reduced as needed, and the steroid was terminated after approximately 10 days (the standard dose is 250 mg/day on ICU days 4-6, and 125 mg/day on ICU days 7-9). In the NP group, each physician could administer 6.6 mg dexamethasone or 1-2 mg/kg mPSL. The standard duration of steroid treatment was 10 days [12].

Data collection

The following information was collected from each patient: age, sex, symptoms, medical history, signs, oxygen saturation, laboratory findings upon ICU admission, and clinical outcomes.

Outcomes

The primary outcome investigated was mortality 28 days after admission. The secondary outcomes investigated were steroid-associated complications within 28 days, including hemorrhagic complications, thrombotic complications, pneumothorax, pneumomediastinum, COVID-19-associated pulmonary aspergillosis, and cytomegalovirus infection. Hemorrhagic complications were defined as gastrointestinal bleeding that required endoscopic hemostasis, cerebral hemorrhage, or intramuscular hematoma. Thrombotic complications were defined as cerebral infarction or pulmonary embolism.

Data analysis

The results were expressed as the median, 25th and 75th quartiles (Q1-Q3) for quantitative data, and numbers and percentages for categorical data. Two-sided Chi-square and Fisher’s exact tests were used to assess the associations between the intervention groups and categorical variables. The Mann-Whitney U test was used to compare continuous variables between the groups. The Kaplan-Meier survival curve was used to estimate the time to death in the intervention groups. The treatment effects in both groups were evaluated using the log-rank test and multivariate Cox proportional hazard model and expressed as hazard ratios (HRs) with 95% confidence intervals (CIs), as appropriate. We performed multiple imputation analyses for missing values [13]. All statistical analyses were performed using SPSS version 28.0 (IBM Corp., Armonk, NY). Statistical significance was defined as a two-sided P-value < 0.05. This manuscript was written according to the STROBE statement guidelines [14].

## Results

Patient characteristics

**Figure 1:**
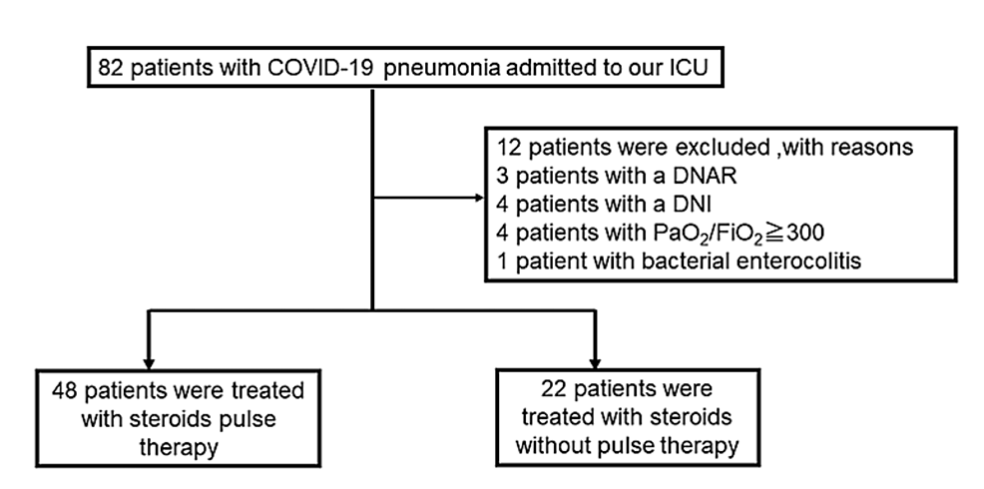
Flow diagram of the study participant selection process, from intensive care unit admission until treatment. Eighty-two patients met the selection criteria. Through the inclusion and exclusion criteria, patients were divided into two groups: patients treated with steroid pulse therapy (n = 48) and patients treated with steroids but without pulse therapy (n = 22). DNAR: Do not attempt to resuscitate; DNI: Do not intubate.

We enrolled 82 patients with COVID-19 pneumonia, who required ICU admission. Of these, 70 patients met our inclusion criteria. The patients were treated with steroid pulse therapy (Group P; n=48) or steroids without pulse therapy (Group NP; n=22) (Figure 1). At baseline, the Acute Physiology and Chronic Health Evaluation (APACHE) II score [15] and interleukin (IL)-6 levels were higher in patients who received steroid pulse therapy (Table 1). Furthermore, the hospital stay and duration of steroid administration were significantly longer in Group P than in Group NP (P=0.01) (Table 2).

**Table 1:** Baseline characteristics of patients at admission to the ICU Data are shown as n (%) or median (interquartile range). PaO2/FiO2: Arterial oxygen partial pressure/fractional inspired oxygen; APACHE: Acute Physiology and Chronic Health Evaluation; KL-6: Krebs von den Lungen-6; PCT: Procalcitonin; IL-6: Interleukin-6; HbA1c: Hemoglobin A1c

	Treated with steroid pulse therapy (n=48)	Treated with steroids without pulse therapy (n=22)	P-value
Age, median	63 (56–74)	73 (59–77)	0.35
Sex, Male (n [%])	38 (79.2%)	20 (90.9%)	0.23
Body mass index (kg/m2), median	25 (22–28)	24 (22–26)	0.17
PaO2/FiO2 ratio	120 (84–160)	119 (100–160)	0.72
APACHE Ⅱ score, median	23 (10–28)	10 (7–20)	0.04
Day from the onset of first symptoms, median	7 (5-9)	8 (5–12)	0.10
Laboratory data			
D-dimer (µg/mL), median	1.1 (0.7–2.1)	1.6 (0.8–3.6)	0.27
FDP (µg/mL), median	3.6 (2.7–4.7)	3.8 (2.8–5.9)	0.64
Lactate dehydrogenase (U/L), median	521 (407–582)	434 (365–542)	0.11
KL-6 (U/mL)	373 (276–564)	442 (297–921)	0.41
PCT (ng/mL), median	0.20 (0.10–0.38)	0.15 (0.09–0.34)	0.51
IL-6 (pg/mL), median	92.2 (55.4–126.6)	54.6 (15.3–84.0)	0.03
HbA1c ≥ 6.5, n (%)	23 (48.9%)（n=47）	8 (40.0%)（n=20)	0.50

**Table 2:** Clinical characteristics of patients Data are shown as n(%) or median (interquartile range).

	Treated with steroid pulse therapy (n=48)	Treated with steroids without pulse therapy (n=22)	P-value
Intubation, (n [%])	36 (75.0%)	10 (45.5%)	0.02
Tracheostomy, (n [%])	11 (22.9%)	3 (13.6%)	0.37
Intensive care unit stay (days) , median	9 (5–14)	6 (3–13)	0.11
Hospital stay (days) , median	20 (15–29)	15 (11–19)	0.02
Steroid therapy duration (days) , median	18 (14–27)	14 (10–19)	0.01
Therapeutic drug			
Methylprednisolone, (n [%])	48 (100.0%)	5 (22.7%)	<0.001
Dexamethasone, (n [%])	0 (0.0%)	16 (72.7%)	<0.001
Remdesivir, (n [%])	48 (100.0%)	20 (90.9%)	0.03
Baricitinib, (n [%])	30 (62.5%)	11 (50.0%)	0.32
Hydroxychloloquine, (n [%])	20 (41.7%)	12 (54.5%)	0.32
Favipiravir, (n [%])	4 (8.3%)	4 (18.2%)	0.23

Primary outcome

**Figure 2:**
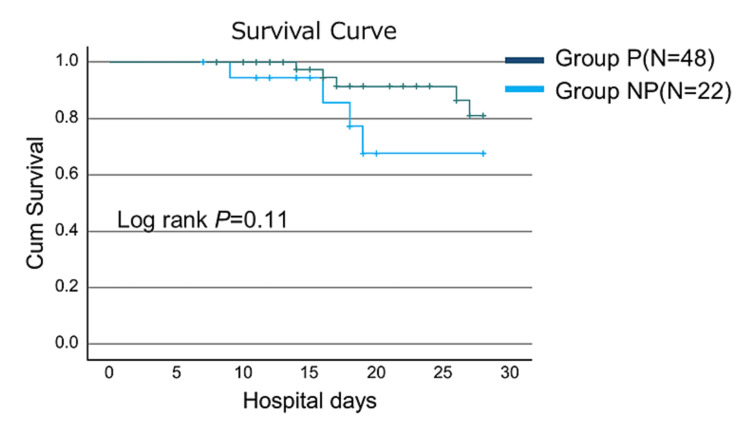
Twenty-eight day cumulative survival curve of the patients in the study No difference in 28-day survival was observed between Group P and Group NP  (log-rank P=0.11).

No difference in 28-day survival was observed between Group P and Group NP (log-rank P=0.11). (Figure 2). In the multivariate Cox proportional hazards model, after adjusting for age, sex, IL-6 level, and APACHE Ⅱ score, treatment with steroid pulse therapy significantly improved the 28-day mortality (HR, 0.14; 95% CI, 0.02-0.86; P=0.03). Multivariate analysis showed that the baseline IL-6 level was significantly associated with mortality, whereas age, sex, and APACHE II score were not (Table 3).

**Table 3:** Factors associated with 28-day mortality HR: Hazard ratio; APACHE: Acute physiology and chronic health evaluation; CI: Confidence interval; ICU: Intensive care unite

	HR	95% CI	P-value
Steroid pulse therapy	0.14	0.02–0.86	0.03
Age	0.994	0.92–1.08	0.88
Sex	0.86	0.08–8.86	0.90
IL-6	1.003	1.001–1.005	0.02
APACHE Ⅱ score on ICU admission	1.07	0.99–1.16	0.10

Secondary outcome

No significant increase in steroid-associated complications was observed during the 28-day follow-up period (Table 4).

**Table 4:** Complication outcomes of patients with COVID-19 COVID-19: Coronavirus disease 2019

	Patients treated with steroid pulse therapy (n=48)	Patients treated with steroids without pulse therapy (n=22)	P-value
Bleeding complications	9 (18.8%)	4 (18.2%)	0.96
Thrombotic complications	7 (14.6%)	2 (9.1%)	0.52
Pneumothorax and pneumomediastinum	9 (18.8%)	7 (31.8%)	0.23
COVID-19-associated pulmonary aspergillosis	3 (6.3%)	1 (4.5%)	0.78
Cytomegalovirus infection	7 (14.6%)	2 (9.1%)	0.52

## Discussion

Our findings suggest that steroid pulse therapy may be effective in patients admitted to the ICU with COVID-19 pneumonia. Our results also suggest that higher IL-6 levels may be associated with increased mortality.

An increasingly critical issue in COVID-19 treatment is how to manage cytokines. Cytokine release syndrome, characterized by increased levels of cytokines (such as IL-6), can cause or worsen acute respiratory distress syndrome (ARDS) and multiple organ failure [16]. Expensive monoclonal antibodies, such as tocilizumab, have been used as therapeutic agents to suppress cytokine release syndrome [17], partially due to an RCT [18] reporting improved survival at 180 days in intubated patients with COVID-19 pneumonia who received an IL-6 receptor antagonist. Based on our results, the suppression of cytokine storms may contribute to reduced mortality.

Steroids are less expensive and are more widely used than monoclonal antibodies. Furthermore, the high mortality rate in patients with severe COVID-19 can be explained by the rapid development of organized pneumonia secondary to SARS-CoV-2 infection [18]. This condition generally requires treatment with high-dose corticosteroids, sometimes referred to as “pulse” doses, for a longer duration [19]. Therefore, steroid pulse therapy may be a realistic treatment option owing to its low cost and widespread use.

There is ample experience in the treatment of interstitial pneumonia with steroid pulse therapy such as mPSL 1000 mg/day. The Japanese guidelines do not recommend steroid pulse therapy for patients with ARDS [20]; however, some facilities in Japan have prescribed steroid pulse therapy for COVID-19 pneumonia [11,21]. The RCTs conducted on patients with COVID-19 pneumonia to date are summarized in Table 5. Three RCTs on patients with COVID-19 pneumonia have reported the efficacy of steroid pulse therapy [4-6]. In a single-blind RCT, Edalatifard et al. [4] reported that the time to clinical improvement and 50-day mortality were lower in the mPSL treatment group (250 mg/day for three days) than in the standard-of-care group (5.9% vs. 42.9%; P<0.001). Pinzon et al. [6] compared the treatment of severe COVID-19 pneumonia with a high-dose mPSL (250-500 mg/day) for three days, followed by oral prednisone for 14 days with 6 mg dexamethasone for 7-10 days. They found that the treatment with a high-dose mPSL significantly decreased the recovery time and ICU admission rates. However, they did not note the differences in overall mortality rates. Farahani [5] demonstrated that the mPSL pulse dramatically improved clinical parameters, including the Glasgow Coma Scale score and oxygen saturation, in patients with COVID-19-related severe respiratory failure. These studies demonstrated the benefits of steroid pulse therapy in patients with COVID-19 pneumonia. However, it is difficult to compare these studies with ours because they were conducted in non-ICU patients with COVID-19 pneumonia.

**Table 5:** Characteristics of previous studies ARDS: Acute respiratory distress syndrome; PaO2/FiO2: Arterial oxygen partial pressure/fractional inspired oxygen

Author	Number of Patients	Severity of COVID-19 pneumonia	Intubated patients at inclusion	Steroid Pulse Dose	Standard Steroid	Primary Outcome
Edalatifard et al. [4] 2020	68	Severe (Noninvasive ventilation 37.1%)	0	Methylprednisolone 250 mg/day for 3 days	Glucocorticoid	The time of clinical improvement or 50 day mortality
Farahani et al. [5] 2020	29	Severe	0	Methylprednisolone 1000 mg/day for 3 days	Prednisolone 1 mg/kg	Mortality
Pinzón et al. [6] 2021	216	Severe (ARDS Evolution Mild 56.5%)	0	Methylprednisolone 250 to 500 mg for 3 days	Dexamethasone 6 mg	The recovery time, the need for transfer to intensive care
Gudino et al. [7] 2022	128	Severe (High-flow oxygen devices, and non-invasive mechanical ventilation are excluded)	0	Methylprednisolone 250 mg for 3 days	Dexamethasone 6 mg	Mortality rate at 28 days.
Salvarani et al. [8] 2022	304	Severe (PaO2/FiO2 ratio≒200 )	0	Methylprednisolone 1000 mg/day for 3 days	Dexamethasone 6 mg	Hospital discharge without the need for supplementary oxygen

Two studies [7,8] reported that steroid pulse therapy did not improve outcomes in patients with severe COVID-19 pneumonia compared with the usual therapy group. In our study, up to 70% of patients were intubated (critically ill patients). The PaO2/FiO2 ratio was approximately 120. Comparing the results of previous studies with ours, the severity of the respiratory disease was greater in our patient group. The results of our study suggest that steroid pulse therapy may be effective in severely ill patients with low PaO2/FiO2.

To improve survival with steroid pulse therapy, it is important to avoid the complications caused by steroid administration. An increased risk of secondary infections has been reported in studies on steroid pulse therapy for patients with early ARDS [22]. However, the number of complications did not increase [23]. Furthermore, although steroid administration in COVID-19 pneumonia can cause adverse effects, the incidence of such events is unknown, owing to differences in the definitions used in previous studies [24]. In our study, the increased incidence of steroid-related COVID-19-associated pulmonary aspergillosis was concerning; however, we found no significant differences between the Groups P and NP.

Hence, steroid pulse therapy may be effective in the treatment of severe-to-critical COVID-19 pneumonia in selected patients. We hope that future studies will further clarify the patient groups for whom steroid pulse therapy is effective.

Limitations

First, the single-center design and short data collection period resulted in a relatively small sample size, which might have influenced the accuracy of our findings. Moreover, the sample size may have been insufficient for the multiple regression analysis. We assumed that the APACHE II score was not a significant predictor of mortality because of small sample size. Further studies with larger sample sizes are required to improve our understanding. Second, there may be many confounding factors between Groups P and NP that have not been examined here, and randomized controlled trials adjusted for patient background are warranted. Third, tracheal intubation was performed based on the judgment of the individual physician. Observational studies using strict intubation criteria are required. Fourth, infectious complications are probably an important factor affecting mortality but were not considered in this study owing to difficulties in defining them. Therefore, improved study designs focusing on infectious complications and mortality associated with steroid pulse therapy are warranted. Fifth, our results may not reflect the effect of steroids alone because the drugs used, in addition to steroids, were changed during the data collection period. Not only the steroid dose but also the combination of drugs, such as antiviral drugs, may have affected the prognosis of the patients. Sixth, the SARS-CoV-2 strain during the data collection period was mainly B.1.617.2 [25]. However, B.1.1.529 was the predominant SARS-CoV-2 strain in 2022 [26], and hence the adaptation of the results of this study is controversial.

## Conclusions

Steroid pulse therapy may improve the 28-day mortality in patients admitted to the ICU with COVID-19 pneumonia. However, the safety and efficacy of steroid pulse therapy need to be demonstrated using RCTs or big data analysis.
